# Efficacy of diversely isolated lytic phages against multi-drug resistant *Enterobacter cloacae* isolates in Kenya

**DOI:** 10.4102/ajlm.v11i1.1673

**Published:** 2022-08-11

**Authors:** Ivy J. Mutai, Angela A. Juma, Martin I. Inyimili, Atunga Nyachieo, Anthony K. Nyamache

**Affiliations:** 1 Phage Biology Laboratory, Institute of Primate Research, Nairobi, Kenya; 2 Department of Biochemistry, Biotechnology and Microbiology, Faculty of Pure and Applied Sciences, Kenyatta University, Nairobi, Kenya; 3 Department of Human Anatomy, University of Nairobi, Nairobi, Kenya

**Keywords:** lytic phages, *Enterobacter cloacae;* multi-drug resistance, nosocomial infections, Nairobi County, Kenya

## Abstract

**Background:**

*Enterobacter cloacae* causes nosocomial infections in 15% of patients in low- and middle-income countries with emergence of carbapenem resistance. The utilisation of bacteriophages for therapeutic purposes is crucial for eradicating these resistant bacterial strains.

**Objective:**

This study evaluated the efficacy of lytic phages on bacterial isolates of *E. cloacae* and determined their stability in various physicochemical conditions.

**Methods:**

Twenty-nine lytic phages were isolated from the waste water of six informal settlements in Nairobi County, Kenya, from July 2019 to December 2020 and cross-reacted with 30 anonymised clinical isolates of *E. cloacae.* Six phages were then selected for physicochemical property studies. Phages were described as potent upon lysing any bacterial strain in the panel.

**Results:**

Selected phages were stable at 4 °C – 50 °C with a 5.1% decrease in titre in four of six phages and a 1.8% increase in titre in two of six phages at 50 °C. The phages were efficient following two weeks incubation at 4 °C with optimal activity at human body temperature (37 °C) and an optimal pH of 7.5. Phages were active at 0.002 M and 0.015 M concentrations of Ca^2+^ ions. The efficiency of all phages decreased with increased exposure to ultraviolet light. All phages (*n* = 29) showed cross-reactivity against anonymised clinical isolates of *E. cloacae* strains (*n* = 30). The most potent phage lysed 67.0% of bacterial strains; the least potent phage lysed 27.0%.

**Conclusion:**

This study reveals the existence of therapeutic phages in Kenya that are potent enough for treatment of multi-drug resistant *E. cloacae.*

## Introduction

*Enterobacter cloacae* is a Gram-negative rod-shaped bacteria that is associated with nosocomial infections.^1^ This organism is naturally resistant to ampicillin, amoxicillin/clavulanic, cephamycin and the first- and second-generation cephalosporins due to chromosomally encoded AmpC β-lactamase.^1^ *Enterobacter cloacae* is a normal flora of the human gastrointestinal tract, which can cause opportunistic infections among immunocompromised individuals, the elderly and newborns.^1,2^ This organism causes septicemia, endocarditis, urinary tract infections, wound infections, and meningitis in newborns.^1,2^ These infections can lead to prolonged hospitalisation and higher cost of treatments, increased antibiotic use and pressure leading to the development of antimicrobial resistance in the hospital. In addition, the rates of morbidity and mortality among critical patients can also increase.^1,2^

*Enterobacter cloacae* causes nosocomial infections that affect at least 7% of patients in high-income countries; such infections are twice as high (15%) in the low- and middle-income countries.^3^ *Enterobacter cloacae* is ranked as the third most predominant organism causing nosocomial infections and the second most predominant carbapenem-resistant organism, according to studies conducted in the United States.^1,4,5,6^ According to Nyangacha et al., *E. cloacae* resistance was observed among patients suffering from tungiasis, or jigger disease, a secondary infection in a study done in the western part of Kenya with the following resistance profiles: ampicillin (75.0%), amoxicillin-clavulanic acid (25.0%), tetracycline (50.0%), ceftazidime (25.0%) and cefuroxime (25.0%).^7^ Globally, the emergence and spread of carbapenemase producers *E. cloacae* has been reported with the prevalence rate of 59.5% being reported in Japan and China in three tertiary hospitals.^8,9,10^ This record of a high rate of drug resistance is a worrying trend for this organism.^10^ There is a growing need to conduct active surveillance of *E. cloacae*, in order to control and prevent further spread of drug resistance to other low-prevalence countries.^8,10,11^

In order to control the current rise in antimicrobial resistance, it is clear that an alternative to the use of antibiotics is urgently needed. Bacteriophages (phages) have been proposed as an alternative to antibiotics based on reported evidence.^12,13^ Bacteriophages are obligate parasites; hence, they infect bacteria and form potential antimicrobial agents capable of killing bacteria, including the drug resistant strains.^11,13,14^ Phages naturally multiply through feeding on the bacteria leading to their lysis which in the process regulates bacterial populations within the ecosystems.^13,14,15^ Since phages are very specific to bacteria, their lysis process could be exploited for the development and production of new therapeutic agents.^11^ Given this revelation, phage hunting is now pursued to aid in disease management, as an alternative to elimination and prevention of multi-drug resistant (MDR) strains.^10,11^

However, *E. cloacae* is a bacteria of medical importance, but there is limited information on the application of bacteriophages as alternative antimicrobial agents against this bacteria.^1,2,5^ Additionally, there is scarce information on studies of lytic phages against *E. cloacae* in Kenya. The efficacy of phages in treatment of disease-causing bacteria has been exhibited by numerous reports. On the other hand, there is scarce information about their physicochemical properties. Furthermore, the stability of Kenyan phages under various extreme conditions of alkalinity, acidity, high and low temperatures, ultraviolet exposure, salinity and storage has not been fully studied. We argue that determining the effect of external factors that could influence the yield and potency of phage preparations is important as one prepares phages as an alternative to antibiotics.

This study, therefore, evaluated the efficacy of lytic phages in vitro on a panel of bacterial isolates of *E. cloacae* and their stability under different physicochemical environments..

## Methods

### Ethical considerations

Ethical clearance was not required for this study, since there were no human subjects or animals models used in this research; however, this study was registered with the Institutional Research and Ethics Committee (ISERC) of the Institute of Primate Research (IPR/IRC/2014). The bacterial isolates used in this study were anonymised clinical isolates obtained from the Kenya Medical Research Institute (KEMRI) Center for Microbiology (SERU#2767), in Nairobi, Kenya. For environmental waste water collection, an approval was issued by Nairobi Water and Sewerage Company (NAWASCO#NCWSC/TRG14/109).

### Sample collection

Environmental waste water collection was done by the Institute of Primate Research Phage Biology Group from July to August 2019. Samples were collected from six informal settlements: Kibera, Dandora, Kariobangi, Huruma, Mathare and Korogocho, all in Nairobi County, Kenya. Three samples per settlement were collected making a total of 18 samples. Dark screw cap containers (to prevent direct light) were used to collect the samples which were transported to the Institute of Primate Research Phage laboratory using cooler boxes as the secondary containment followed by storage at 4 °C and processed within three weeks. The three samples per settlement were named using number designations in the following order: 1, 2 and 3.

### Bacterial isolation and identification

An MDR isolate of *E. cloacae* isolated from environmental waste water was used for phage isolation. This bacterium was identified using culture media, microscopic and biochemical examination using Vitek II machine (BioMérieux, Marcy-L’Etoile, France) for identification and antimicrobial susceptibility of bacteria.^16^ A panel of anonymised clinical bacterial isolates (not evaluated for antimicrobial susceptility test) of *E. cloacae* (*n* = 30) were subjected to the phages to assess cross infectivity.

### Phage isolation

Phages were isolated through an enrichment method according to the methods described by Akhtar et al. and Clokie et al. with slight modifications.^17,18^ Briefly, 30 mL of each environmental waste water was centrifuged at 10 000 × gravitation for 10 min (Centrific™, Centrifuge Fisher Scientific, Waltham, Massachusetts, United States). The supernatant was mixed with an equivalent volume of tryptose soy broth (TSB) (HiMedia, Mumbai, India), and inoculated with 1 mL of 18 h-old MDR *E. cloacae* culture. The mixture was incubated overnight at 37 °C in a shaker incubator at 120 rotations per minute (Lab-line^®^ Incubator-Shaker, Waltham, Massachusetts, United States). The cultures were then centrifuged at 10 000 × gravitation for 10 min, and the supernatant sterilised using a 0.22 µm syringe filter (Millipore, Merck, Darmstadt, Germany) and stored at 4 °C for use in the spot test.

### In vitro screening for phages (spot testing)

Phages were screened through spot test procedures according to Clokie and Kropinski (2010) with slight modifications.^18^ Briefly, a lawn of 24 h-old MDR *E. cloacae* isolate (100 µL) was prepared in soft agar (0.7%) with TSB on a tryptose soy agar (TSA) plate (HiMedia, Mumbai, India). Ten-fold serial dilutions of the phage filtrate were prepared and 5 µL of each dilution was spotted on a well-labelled plate with dilutions ranging from 10^−1^ to 10^−8^ followed by overnight incubation at 37 °C. Observation of plaques (clear-patched regions) on the bacterial lawn was recorded as positive for the phage. For plates with distinct plaques, the well-isolated plaques were harvested using a sterile Pasteur pipette and suspended in 200 µL sterile saline magnesium (SM) buffer (100 mM sodium chloride, 10 mM magnesium sulphate, 50 mM Tris-HCl, pH 7.5 and 0.01% weight by volume gelatin), vortexed and incubated at room temperature for 1 h before centrifuging at 4000 gravitation for 5 min to remove any remaining debris, labelled and stored at 4 °C.

### Preparation and titration of phage lysate (plaque assay)

For plates without well-isolated plaques, the double agar overlay method was employed with slight modifications.^19^ Briefly, 100 µL of the 10-fold serial dilution of the lysate (from the spot with the least number of plaques) was mixed with 100 µL of 18 h-old *E. cloacae* in 6 mL molten agar (0.7% agar with TSB) and dispensed on TSA plate (1.5% agar with TSB) medium and allowed to solidify before incubation at 37 °C for 18 h. Well-isolated plaques were identified and marked before harvesting each plaque using a sterile Pasteur pipette, vortexed and incubated at room temperature for 1 h before centrifuging at 4000 gravitation for 5 min to remove any remaining debris, labelled well and stored at 4 °C.

### Calculation of phage titre

In plates with distinct plaques for the spot test and plaque assay, the plaques were counted respective to their dilution factors employing the following formula:

Phage titre (pfu/mL)=plaques per plate×volume plated (mL)×dilution factor. [Eqn 1]

^18^

### Effect of temperature on phage titre

Phage adsorption rates on the host bacterium were recorded at the temperatures 4 °C, 10 °C, 20 °C, 37 °C and 50 °C. Then 100 µL of actively growing host strain cultures in TSB to an optical density of 600 nm (OD600) of 0.6 was used to make an overlay on TSA plates. The selected phages were then incubated at these different temperatures for 1 h and then placed at room temperature (20 °C – 27 °C) for 30 min prior to performing spot tests. The outcomes were given as a log_10_ of phage titre.^20,21,22^

### Effect of pH on phage titre

The effect of pH 2, 5, 7, 9, 11 and 13 on phage titre and viability of phages was studied in TSA plates by the spot test method.^18^ The SM buffer was adjusted to the desired pH by the use of NaOH and HCl. The actual pH of the SM was determined with a pH meter (Hanna Instruments Inc. Woonsocket, Rhode Island, United States) and this was used as a control or standard. The adjusted SM buffers were used for serial dilutions of the phages under study. Dilutions of the phage stocks were done to get the working dilution factors. Roughly, 10^6^ pfu/mL of 20 µL phages (individual phages) was added to 180 µL of SM buffer, after an earlier adjustment of pH (2–11), in Eppendorf tubes, followed by 30 min of incubation at 37 °C. The remaining phages were determined by spotting the phages in different pH and the outcomes indicated as pfu/mL.^23^

### Influence of Ca^2+^ ions on phage titre and phage stability

The effect of the divalent cations on bacterial lysis and phage adsorption was investigated by varying the concentration of CaCl_2_: 0.000 M, 0.005 M, 0.010 M and 0.015 M in soft agar during preparation. Plaque assays were conducted in duplicate, followed by overnight incubation at 37 °C. Phage titre was determined at the different salt concentrations as log_10_ and comparisons made with the increase in salt concentrations.^21^

### Storage stability of *Enterobacter cloacae* phages

Stability of the phages during storage was investigated using a previously described method with slight modifications.^24^ Briefly, 3 mL of the selected phages with known phage titre were aliquoted into 15 mL centrifuge tubes and wrapped with aluminium foil to prevent direct light and kept at –20 °C, 4 °C, and 37 °C for two weeks. The spot test method was used to determine the effectiveness and the efficiency of the phages after storage.

### Effect of ultraviolet light on phage titre

The spot test method was used to determine the effect of ultraviolet light on phage irradiation with various modifications.^25^ Briefly, 10 µL of each phage of known titre from each site was aliquoted into five sterile PCR tubes and labelled as 0, 5, 10, 15 and 20 min of ultraviolet light exposure. The phages for each time period were placed in a Biosafety Cabinet Level 2 (290 nm – 320 nm, BSC-2, Haier, Tokyo, Japan) and the ultraviolet light turned on for the required time; the phages were then removed simultaneously. Polymerase chain reaction (PCR) tubes have been found to permeate ultraviolet light rays and affect the integrity of DNA; hence, the tubes were capped during this study.

### Host range determination of phages

To ensure the specificity of bacteriophages, their effect on other bacterial genera and species was investigated. The anti-bactericidal efficacy of individual phages (*n* = 29) was evaluated through the spot test method against each individual *E. cloacae* isolate (*n* = 30) as described by Kutter (2009).^23^ *Staphylococcus aureus*, a bacteria from another genera, was used as negative control. A volume of 5 µL of individual phage stock was spotted on a TSA plate with a lawn of 100 µL overnight cultured host bacteria in soft agar, which was examined for bacterial lysis after 18 h – 24 h. The spot tests were performed in duplicate. A clear zone was considered as a positive infection in the tests and negative with no cross-reactivity.^26^ A phage was termed ‘potent’ upon lysing any bacterial strain in the host range panel. A phage with the widest spectrum of lysis activity on the tested bacterial strains was termed the ‘most potent’, while a phage with the lowest spectrum of activity on the tested bacterial strains was termed the ‘least potent’. A bacteria that was sensitive to a phage infection was termed ‘susceptible’.

### Data analysis

All the experiments were performed in triplicate and the mean values obtained. Statistical entry was carried out and given treatment in Microsoft Excel 20 for Windows (Microsoft, Redmond, Washington, United States) and Epidemiological Information (EPI info7^TM^, Centers for Disease Control and Prevention, Atlanta, Georgia, United States). One-way analysis of variance using Statistical Package for Social Sciences version 20 (SPSS Inc., Chicago, Illinois, United States) was used to determine significant differences at *p* < 0.05. The surviving phage population obtained in each study were converted to log_10_ PFU/mL. Data presentation was performed using GraphPad Prism version 5.00 for Windows (GraphPad Software, San Diego, California, United States).

## Results

### Antimicrobial susceptibility profile of the host bacterium (*Enterobacter cloacae*)

This isolate was resistant to five classes of antibiotics namely: beta-lactams, penicillins (ticarcillin or clavulanic acid, and piperacillin), cephalosporins (cefuroxime, cefuroxime axetil, ceftriaxone, and cefepime), monobactams (aztreonam), chloramphenicols (amphenicol), tetracycline (tetracycline, minocycline), quinolones (levofloxacin) and sulphonamides (trimethoprim). It was susceptible only to: meropenem (carbapenem) and tigelcycline (glycylcycline).

### Isolated phages

A total of 29 phage strains were isolated, with four phages from Dandora, five phages from Huruma, five phages from Kibera, five phages from Kariobangi, seven phages from Korogocho and three phages from Mathare. These phages were named using number-letter designations according to the sample source. For example, from Kibera 1 settlement: the phages were named Kibera 1a, 1b, and 1c. All the phages were subjected to host range studies. From the 29 phages isolated from the six sources, one phage from each source that had complete lytic properties and a lytic zone diameter of ≥ 3 mm was selected for study of physicochemical properties.

### Effect of temperature on phage titre

**FIGURE 1:**
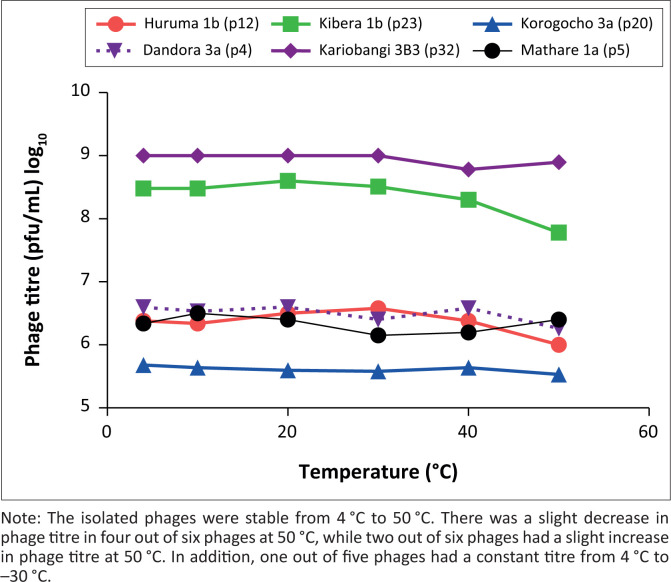
Effect of temperature on phages isolated in Nairobi County, Kenya, July 2019 to December 2020. Phages were named based on source and phage strain number for example Huruma 1b (p12) means Huruma 1b source; phage strain number 12.

The isolated phages were stable from 4 °C to 50 °C (Figure 1). There was a slight decrease in phage titre in four out of six phages at 50 °C, and two out of six phages had a slight increase in phage titre at 50 °C. In addition, one out of five phages had a constant titre from 4 °C to 30 °C.

### Effect of pH on phage titre

**FIGURE 2:**
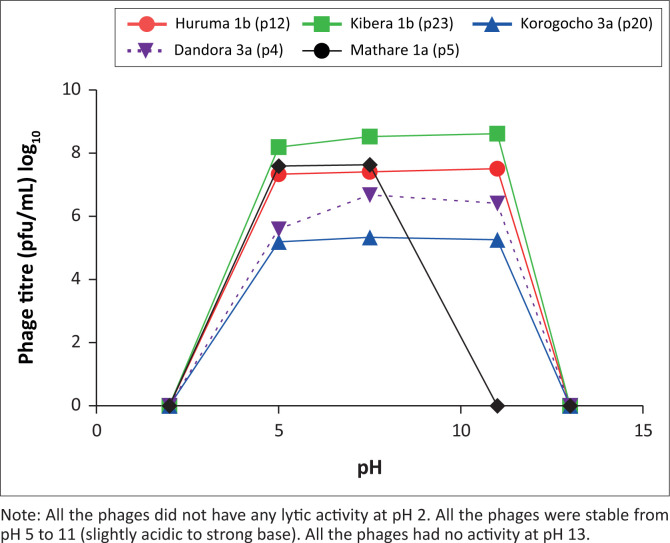
Effect of pH on phages isolated in Nairobi County, Kenya, July 2019 to December 2020. Phages were named based on source and phage strain number for example Huruma 1b (p12) means Huruma 1b source; phage strain number 12.

No phages had any lytic activity at pH 2. All the phages were stable from pH 5 to 11 pH (slightly acidic to strong base). No phages had activity at pH 13 (Figure 2).

### Influence of Ca^2+^ ions on phage titre and phage stability

**FIGURE 3:**
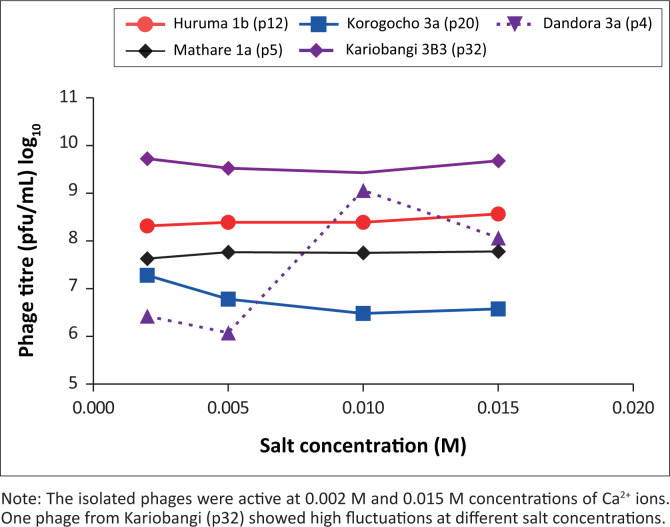
Effect of salt on phages isolated in Nairobi County, Kenya, July 2019 to December 2020. Phages were named based on source and phage strain number, for example Huruma 1b (p12) means Huruma 1b source; phage strain number 12.

The addition of calcium chloride (0.002 M – 0.015 M) salt increased the adsorption rate and phage titre of phages in three out of five of the phages and a decrease in two out of five phages (Figure 3).

### Storage stability of *Enterobacter cloacae* phages

**FIGURE 4:**
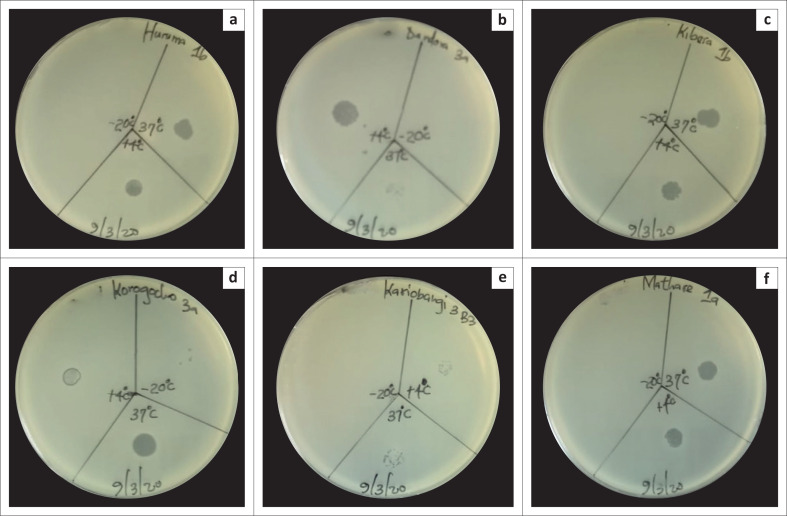
Effect of storage on phage effectiveness and stability isolated in Nairobi County, Kenya, July 2019 to December 2020. The plates show the stability and lysis activity of: (a) Huruma 1b phage, (b) Dandora 3a phage, (c) Kibera 1b phage, (d) Korogocho 3a phage, (e) Kariobangi 3b3 phage, and (f) Mathare 1a phage at 4 °C, 37 °C and −20 °C following 2 weeks of storage. These phages were isolated in Nairobi County, Kenya, between July 2019 and December 2020. The phages were stable and efficacious at 4 °C and 37 °C with minimal or no activity at −20 °C.

All the phages were stable and efficacious at 4 °C and 37 °C. There was minimal or no activity at −20 °C following two weeks of storage (Figure 4).

### Effect of ultraviolet light on phage titre

**FIGURE 5:**
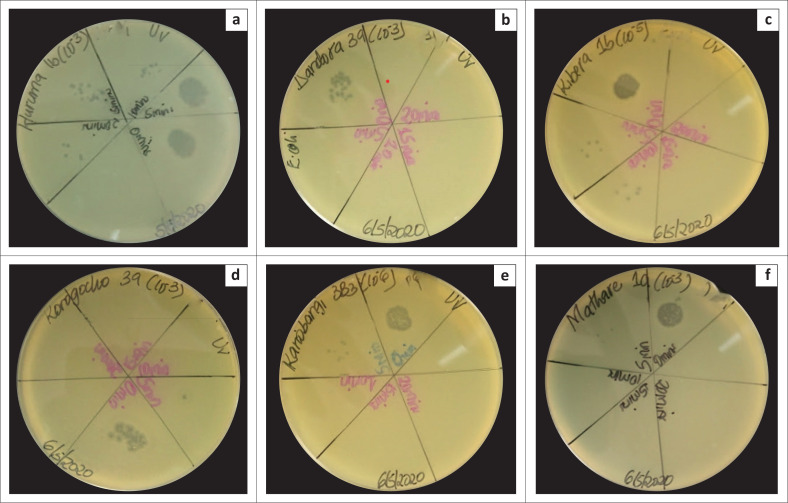
Effect of ultraviolet light exposure on phage survivability isolated in Nairobi County, Kenya, July 2019 to December 2020. The plates show the effect of ultraviolet light exposure from 0 min, 5 min, 10 min, 15 min and 20 min on: (a) Huruma 1b phage, (b) Dandora 3a phage, (c) Kibera 1b phage, (d) Korogocho 3a phage, (e) Kariobangi 3b3 phage, and (f) Mathare 1a phage isolated in Nairobi County, Kenya, between July 2019 and December 2020. The selected phages had decreased activity with increase in time from the 5th min to the 15th min, and no activity at all at the 20th min because all the phages had been destroyed.

Exposure to ultraviolet light (290 nm – 320 nm) resulted in decreased phage titre from the 5th min to the 15th min. At the 20th min, there was no activity at all, which indicated that all the phages had been sterilised (Figure 5).

### Host range spot test analysis on *Enterobacter cloacae* bacterial isolates

**FIGURE 6:**
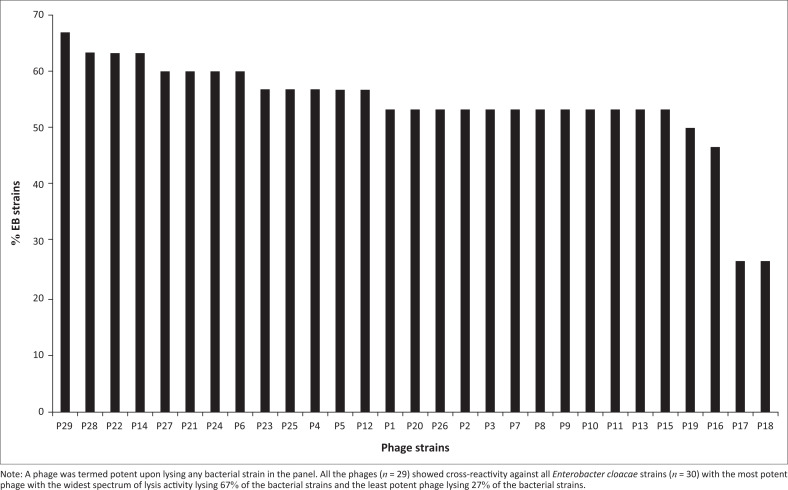
Percentage potency of isolated phages on *Enterobacter cloacae* strains isolated in Nairobi County, Kenya, July 2019 to December 2020.

**FIGURE 7:**
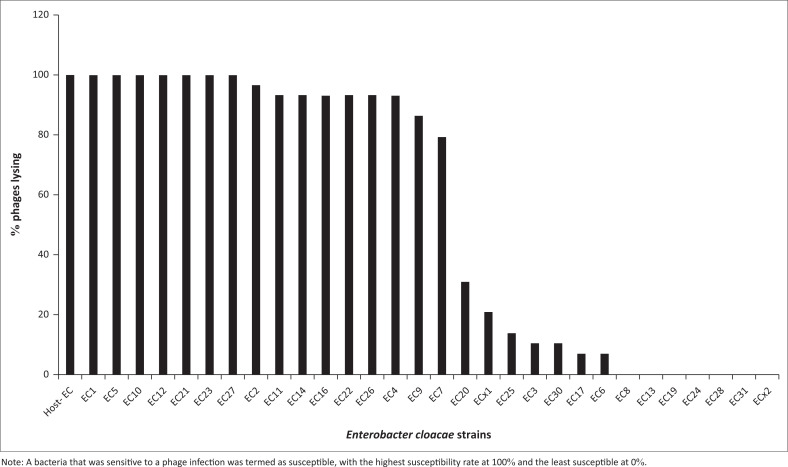
Susceptibility of *Enterobacter cloacae* bacteria to isolated phages in Nairobi County, Kenya, July 2019 to December 2020.

All the isolated *E. cloacae* phages (*n* = 29) showed cross-reactivity against *E. cloacae* strains (*n* = 30) (Figure 6). In order of individual phage potency from the highest to the least, the reactivity levels were: 67% (1 phage), 63% (3 phages), 60% (4 phages), 57% (5 phages), 53% (12 phages), 50% (1 phage), 47% (1 phage) and 27% (2 phages). According to the most susceptible bacteria to phages from the highest to the least, the following data were obtained: 100% (8), 97% (1), 93% (6), 86% (1), 79% (1), 31% (1), 21% (1), 14% (1), 10% (2), 7% (2) and 0% (7) (Figure 7).

## Discussion

This study found that the *E. cloacae* isolates from environmental waste water were resistant to five classes of antibiotics and hence termed as a MDR organisms. This is an indication that our environment habours MDR isolates that could be pathogenic to human and animal health. The antimicrobial profile of this isolate made it a good candidate for phage isolation.

All of the isolated *E. cloacae* phages showed cross-reactivity against *E. cloacae* strains with the most potent phage lysing 67% of the bacterial strains and the least potent phage lysing 27% of the bacterial strains. A bacteria that was sensitive to a phage infection was termed as susceptible, with the highest susceptibility rate at 100% and the least susceptible at 0%. These results have similarities with previous studies done in India in 2019, on the host range of *E. cloacae* phage, which was able to lyse four species of the bacteria.^27^ Additionally, in a study done in Portugal in 2015, the use of three phages of *E. cloacae* as a cocktail for inactivation of urinary tract infections increased the potency of the phages in killing MDR *E. cloacae.*^28^ This specificity of the phages to the host bacteria is being exploited for therapeutic purposes in the treatment of various MDR bacteria.^29^ The specificity of the lytic activity is a characteristic that has been utilised for the development and production of novel therapeutic agents.^11^ Host-range specificity in phage therapy is one of the major advantages for its success while it spares the commensal microbes from destruction during remedy.^30^ The specificity of the phages to their host bacteria is attributed to the phage host receptors involved in recognition, interaction and adsorption during attachment.^31^ Additionally, the receptors are recognised by the ends of the virion’s long tail fibres of the phage towards the host bacteria.^32^

The stability of the *E. cloacae* phages obtained from this study varied from 4 °C to 50 °C. This stability concurs with *E. cloacae* phages previously isolated in Lahore, Pakistan.^33^ Additionally, the phage titre fluctuated with different temperature conditions: there was a slight decrease in phage titre in four of six phages at 50 °C while two of six phages had a slight increase in phage titre at 50 °C. In addition, one of five of the phages had a constant titre from 4 °C to 30°C. The observed variation was also observed in a study done in Iowa, United States, with the yield of phages being highly dependent on temperature.^34,35^ The elucidation of phage stability at different temperatures is needed to establish phage effectiveness as alternative therapeutic agents.

From our pH stability studies, it is evident that the isolated *E. cloacae* phages were not stable in very acidic environments such as pH 2. This could be associated with the denaturation of the phage protein coat at low pH and stability being attained at basic pH above 5, with optimal activity in pH-neutral conditions (pH 7.5).^36^ However, the inactivation and reduction of the lytic activity of the phages decreased in high alkaline conditions (pH 11–13). This could be attributed to dissociation of the capsid protein due to high concentrations of hydrogen and hydroxyl ion in the solution.^36^ These findings concur with findings from previous studies done in Portugal and Poland with optimal phage stability at neutral pH (7.5).^24,37^ The ability of these phages to survive in the neutral pH (7.5) could be exploited or utilised in various applications such as sterilisation of hospital equipment, industrial mass production and for therapeutic purposes in patients with MDR infections of *E. cloacae*.

In our study, there was increased activity of the isolated phages in 0.002 M and 0.015 M concentrations of Ca^2+^ ions. But there was a slight drop of phage activity (1.79% drop) at 0.05 M Ca^2+^ concentration in four out of six6 phages. In a study done in Ireland in 2015, calcium was found to accelerate the phage lytic cycle with an impact on dairy fermentations.^38^ Some phages require the cation for nucleic acid injection, efficient adsorption to cell wall binding sites and enhanced stability.^38,39^ The addition of the salts in our study corresponded with the above findings. Salt availability also aids in the penetration processes of the phage genome into the host cytoplasm.^39^ A slight drop in phage titre of some phages might have been caused by the increase in growth of phage aggregates that might have resulted from neutralisation of the negatively charged moieties on the phage surface by cation binding with an increase in calcium salt concentration.^40^

All the phages in the current study were stable and efficacious at refrigerated temperature (4 °C) with optimal activity at human body temperature (37 °C) and minimal or no activity after being frozen (−20 °C) for two weeks. The crystal structure of ice destroys phages at −20 °C; hence, this storage is highly discouraged.^41,42^ The viability of phages at 4 °C has also been reported in other studies.^24,37,43^ A 5% – 10% glycerol addition to the phage suspension possibly warrants safe viability and infectivity for 30 days or longer-term storage at −20 °C or −70 °C.^44^

Ultraviolet light is known to kill viruses and bacteria cells by disrupting their DNA by damaging the thymine bases through creating a reaction between molecules or creating dimers.^45^ In our study, phages had decreased activity after exposure to ultraviolet light, and efficiency and efficacy decreased upon continuous sterilisation up to the 15th min and no activity at the 20th min. This effect of irradiation has also been reported in previous studies done in the United States in 2002 and 1947.^25,46^ In addition, a study conducted in China in 2020 reported that ultraviolet light potentially reduced phage titres in pathogen reduction quality.^47^

### Limitations

In host range determination, a panel of 30 anonymised clinical isolates of *E. cloacae* and one *Staphylococcus aureus* (control) were used for cross-reactivity studies. However, no antimicrobial susceptibility studies for these bacterial isolates were carried out.

### Conclusion

This study reveals the existence of the most potent lytic phages that are effective on MDR *E. cloacae* isolates found in Kenya. The existence of diverse phage strains from the sampled areas provided an effective cocktail of phages that could be used as antimicrobial agents. Findings from this study demonstrate that physicochemical properties influence the efficacy of phages in their antimicrobial activities and are worth considering.
